# COPE: C*ommunity* O*utreach* & P*rofessional* E*ngagement* – a framework to bridge public mental health services with religious organizations

**DOI:** 10.3389/fpsyt.2025.1461804

**Published:** 2025-08-08

**Authors:** Glen Milstein, Joseph M. Currier, Charles Dent, Melissa McKnight, David Eckert, Amy Manierre

**Affiliations:** 1 Department of Psychology, The City College of New York (CUNY), New York City, NY, United States; 2 Psychology Department, University of South Alabama, Mobile, AL, United States; 3 Charles Dent Enterprises, Mobile, AL, United States; 4 Veterans Recover, Mobile, AL, United States; 5 Intersect Division of Access Services, Fort Washington, PA, United States; 6 Emmanuel United Church of Christ, Sebring, FL, United States

**Keywords:** community and collaboration and continuity, social determinants and social support, religion and spirituality and faith, substance use disorder, wellbeing and wellness, mental disorder, recovery, relapse

## Abstract

The primary purpose of public mental health is to promote wellbeing. The World Health Organization (WHO) and the Substance Abuse and Mental Health Services Administration (SAMHSA) have found that it is crucial to engage community to improve wellbeing and to support persons at times of stress. The United States Surgeon General has reported on significant debilitation caused by an epidemic of loneliness, contributed to by the loss of social connections through fewer and less vibrant social infrastructures. WHO, SAMHSA and the Surgeon General recognize that spiritual/faith-based organizations (SFBOs) are prevalent social infrastructures, dispersed geographically, as well as found across diverse economic, ethnic, immigrant, well-served and underserved communities. Because of their prevalence and social connectedness, what role could SFBOs play to improve social cohesion and individual wellbeing, increase community support, reduce dysfunction and sustain recovery? How could mental health service organizations (MHSOs) engage SFBOs in collaborative care? This paper will review evidence that supports the role of religion and spirituality (R/S) to both promote wellbeing, as well as to respond to stressors in ways that can both prevent the onset of mental disorders and support recovery after clinical treatment. We also review negative attributes of R/S that can be the source of trauma and also impede access to mental health care. We provide a framework for C*ommunity* O*utreach* & P*rofessional* E*ngagement* (COPE) to guide collaborations that originate in MHSOs and reach out to SFBOs to build relationships that can become partnerships. Key principles of COPE are to recognize that community and clinic are separate domains, that clergy have both religious and cultural expertise pertinent to wellbeing and social support, and that clinicians have expertise pertinent to assessment and treatment for dysfunction. COPE is a framework to bridge these separate domains in order to facilitate community-engaged collaborative care, which is clinically crucial for persons with more severe mental illness or substance abuse to sustain their recovery. We provide case examples of the COPE categories of collaboration, and include recommendations for future research in the context of outcomes for public mental health.

## 1 Introduction

The primary purpose of public mental health is to promote wellbeing (1–3). Both the World Health Organization (WHO) through its Sustainable Development Goals (SDGs) – as well as the Substance Abuse and Mental Health Service Administration (SAMHSA) through its categories of wellness (4, 5) – have articulated attributes of wellbeing. The WHO and SAMHSA’s wellbeing attributes emphasize social belonging, emotional connectedness, intellectual pursuits and spiritual meaning, along with health, financial stability, and justice. WHO, SAMHSA, and diverse public health and clinician-researchers (6–19), all advocate that in order to promote wellbeing and support, as well as clinical treatment and sustained recovery, mental health systems require partnerships with community infrastructures (i.e. organizations that facilitate social connectedness and support) (5, 20–24).

One robust path to achieve these collaborations is to cultivate partnerships between mental health service organizations (MHSOs) and spiritual/faith-based organizations (SFBOs) (23, 25–29). Since its inception, the WHO has promoted the utility of partnerships between MHSOs and SFBOs (22, 30). In this paper we review evidence that supports the role of religion and spirituality (R/S) to promote wellbeing, as well as respond to stressors in ways that can both prevent the onset of mental disorders and support recovery after clinical treatment (31). We also review negative attributes of R/S that can be the source of trauma and also impede access to mental health care and sustained recovery (32).

Religion and spirituality (R/S) are core domains of identity and psychosocial functioning for most persons (33, 34). A compelling evidence base highlights the role of R/S in preventing the onset of mental ill health and disorders as well as providing resources for recovery and growth when such conditions occur (33, 35). Advances in translational research from university to community affirm the value of assessing peoples’ R/S and – when relevant – including these beliefs, practices, and relationships in psychotherapeutic interventions (35), which include those negative R/S experiences that can be sources of trauma (32).

The salience of a focus on community collaboration is also supported by data that demonstrate that religious practice in a religious community is most predictive of positive health outcomes (33, 35). For example, one study explicitly compared personal vs. communal (congregation-affiliated) religiosity, and observed that personal religiosity alone was not protective: greater personal religiosity – at a low level of communal religiosity – led to negative religious coping, which was significantly associated with grief severity (36).

Consistent with current clinical emphases on whole-health and person-centred care, asking about persons’ R/S can tailor evidence-informed prevention and clinical interventions so as to enter into dialogue with each person’s potent beliefs, values, practices, and relationships. However, these public mental health resources are frequently overlooked by MHSOs, with the result that – even when salient – it is uncommon for MHSOs to enact evidence-informed assessment and integration of R/S in treatment. This is not surprising as mental health providers infrequently receive training in the clinical applications of R/S (37, 38). Therefore, a core ethical challenge for MHSOs, is to establish training and systems of intervention that are capable of attending to needs of persons across the wide variety of their lifespan religious practices and beliefs, as well as persons with no beliefs or practices of a spiritual nature, and persons for whom religion is a source of harm (23).

**Figure 1:**
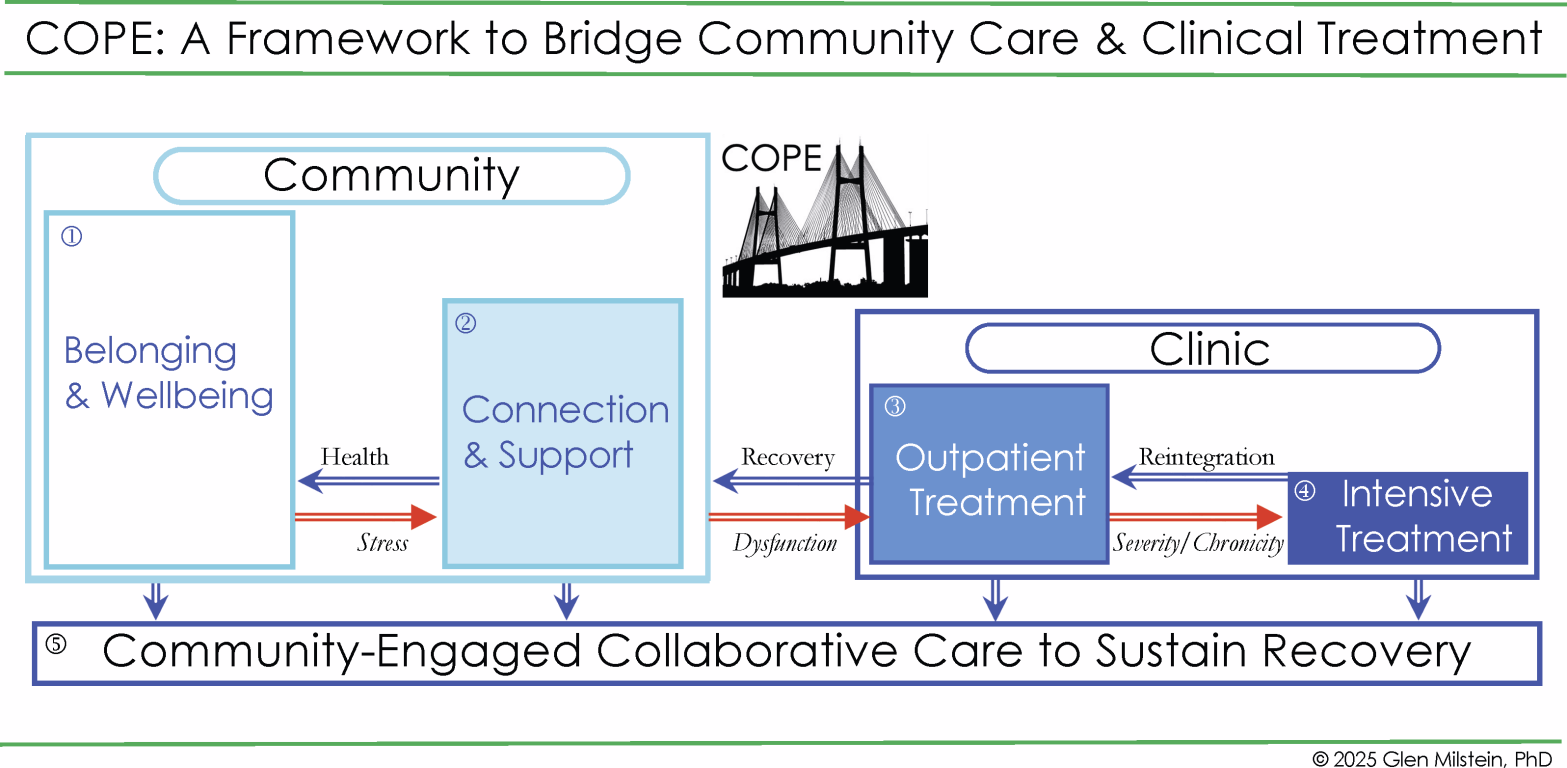
COPE: Framework for collaboration between community care & clinical treatment.

We present a framework (**Figure 1**) of C*ommunity* O*utreach* & P*rofessional* E*ngagement* (COPE) that maps how to collaborate and bridge the resources and goals of communities and clinics to promote wellbeing, prevent dysfunction, provide treatment and sustain recovery (23, 28, 37, 39–46). In turn, we describe examples of COPE in community settings. We conclude with suggestions for future designs of outcomes research for these initiatives. We hope to offer an actionable guide for mental health clinicians and researchers – as well as clergy and lay leaders – to build sustainable and reciprocally beneficial partnerships between MHSOs and the SFBOs in their communities.

## 2 Assessment of policy/guidelines options and implications

### 2.1 An epidemic of loneliness

Recently, the United States Surgeon General has reported on the significant debilitation of wellbeing caused by an epidemic of loneliness (5). Contributing to this malaise is the loss of social connections from ever fewer and less vibrant social infrastructures in our communities (47–49). The Surgeon General also notes that spiritual/faith-based organizations (SFBOs) remain prevalent, and are de facto community resources that may ameliorate loneliness and improve wellbeing. However, these prevalent community resources, are frequently overlooked by clinical practitioners and researchers (5).

### 2.2 Spiritual/faith-based organizations as public mental health resources

The idea of SFBOs as de facto public mental health resources is not new. In “The Churches and Mental Illness” (50), written in 1962 for the Joint Commission on Mental Illness and Health, Richard McCann describes examples of *wellbeing*, promoted by SFBOs through de facto primary prevention:

The “primary level” of prevention, while a profoundly complicated matter, would see the clergy facilitating the maximum contribution of religion to the growth of *whole, mature persons – creative, adventuresome, able to give and receive love fully, with long-range orientations and aspirations* [emphasis added] (p, 239).

Spiritual/faith-based organizations (SFBOs) are naturally-occurring social infrastructures and conduits of prevention and recovery in communities. Religious communities and their leaders are part of the network of community providers who help both promote wellbeing and prevent illness, in well-resourced as well as in underserved communities (2, 29, 30, 51). Persons from under-represented ethnic groups who often contend with other social determinants of poor health (e.g. systemic racism, poverty) (52, 53), as well as older persons – who often have higher disease burdens and poorer health outcomes – tend to be more likely to participate in community SFBOs activities as well as seek help and counsel there (54, 55). Immigrants are also a group in which many people adhere to strong religious beliefs and practices (56–59), and for whom mental health care is often both hard to access and avoided due to stigma (60–62). Therefore, SFBOs have the potential to provide much-needed extra support for all these groups; a useful resource in the drive to “level-up” and improve mental health indices in disadvantaged populations (2, 21, 28, 30, 63, 64). In turn, collaboration between MHSOs and SFBOs could strengthen the range and duration of the support that each offer (18, 52, 53).

### 2.3 Public mental health and religion: structural & cultural bridges

**Figure 2:**
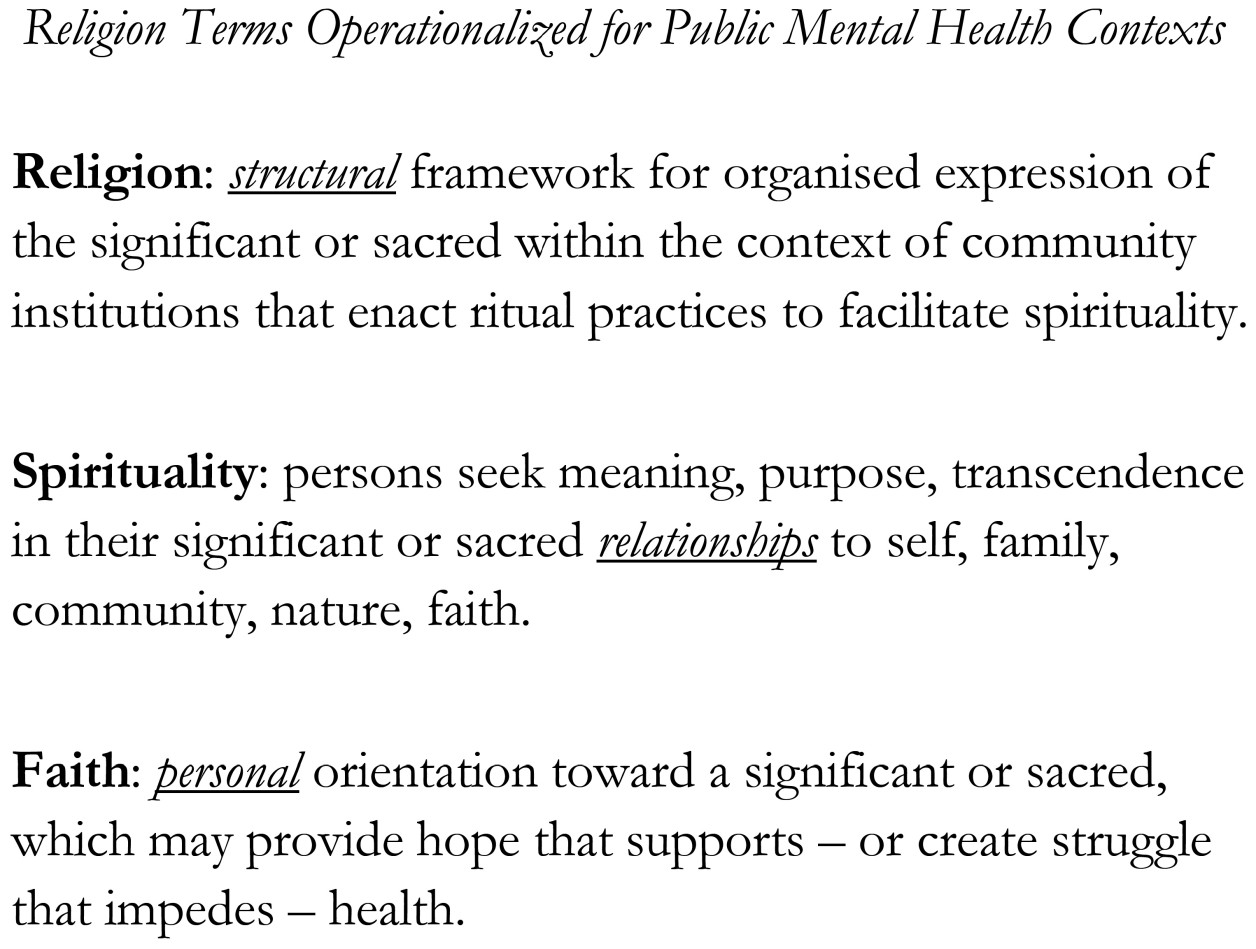
Religion terms operationalized for public mental health contexts.

Religions across the world share two attributes that can organize efforts to bridge community and clinic (**Figure 1**): (1) Religions are structural: they have buildings in locations where followers gather; (2) Religions are cultural and spiritual: their beliefs, rituals and prayers are as varied as the languages of humanity (65–68). The terms Religion, Spirituality and Faith are operationalized for public mental health contexts in **Figure 2** (69–72).

To initiate dialogue with SFBOs, an MHSO will first need to physically locate SFBOs structures. Congregations vary greatly in size and accessibility. Persons from MHSOs who reach out to SFBOs, will also need to learn and respond to the varied cultural beliefs and practices of different denominations, which will guide priorities for how MHSO representatives will interact with the larger community (21, 23).

A core ethic of COPE is the empirical understanding that, religion is ***real***: it is present across humanity and influences both emotions and behaviours (73). This is distinct from any conversation about whether any one religion (or denomination) may be ***true***: that refers to an individual’s or community’s own spiritual path toward specific beliefs of transcendence (**Figure 2**). With this understanding that religion is ***real***, even MHSO clinicians and SFBO clergy and congregants who have divergent worldviews can nevertheless collaborate to reduce the pain caused to individuals and families by mental illnesses and substance abuse. 

#### 2.3.1 Structural bridges: religions construct buildings

The robust public mental health opportunities that are possible when we facilitate sustained interactions of MHSOs and SFBOs are to be expected when one considers that – in addition to untold numbers of unaffiliated spiritual communities – research has identified 356,642 SFBOs across the United States (74). As examples of SFBOs’ prevalence by population densities: Arkansas – with the highest – has an SFBO for every 405 people in the state, and even Nevada – with the lowest – has an SFBO for every 2,016 people. From a community integration and access perspective, no other single category of social infrastructure has so much reach into such a variety of communities, or is present for individuals across their lifespans from infancy to old age. SFBOs are rooted in a variety of communities: those with numerous resources, as well as those with few resources and poor access to care. Clergy and other faith leaders are frequently called on to assist with the material sustenance of the congregants in their communities. SFBOs also frequently assist persons in the community who are not affiliated with their congregation. Particularly in underserved and highly religious locales, SFBOs fill gaps and expand resources for housing, food, counselling, and other supports, which mitigate multiple social determinants of poor mental health (75).

##### 2.3.1.1 Public mental health of attendance

After more than a quarter century of empirical mental health research on the risk and protective factors of religion and spirituality (R/S), rigorous studies have found that attendance at religious services has consistently predicted positive mental health outcomes (33, 34). A recent systematic assessment examined 8,946 empirical research studies of R/S variables in association with serious illness and another 6,274 empirical studies of health outcomes. A multi-stage review determined the highest quality studies with the least evidence of bias. This yielded 441 studies of serious illness and 276 studies of health outcomes. The most consistent and robust findings were the benefits of communal religiousness factors such as religious service attendance on physical, mental and substance use outcomes (35).

**Figure 3:**
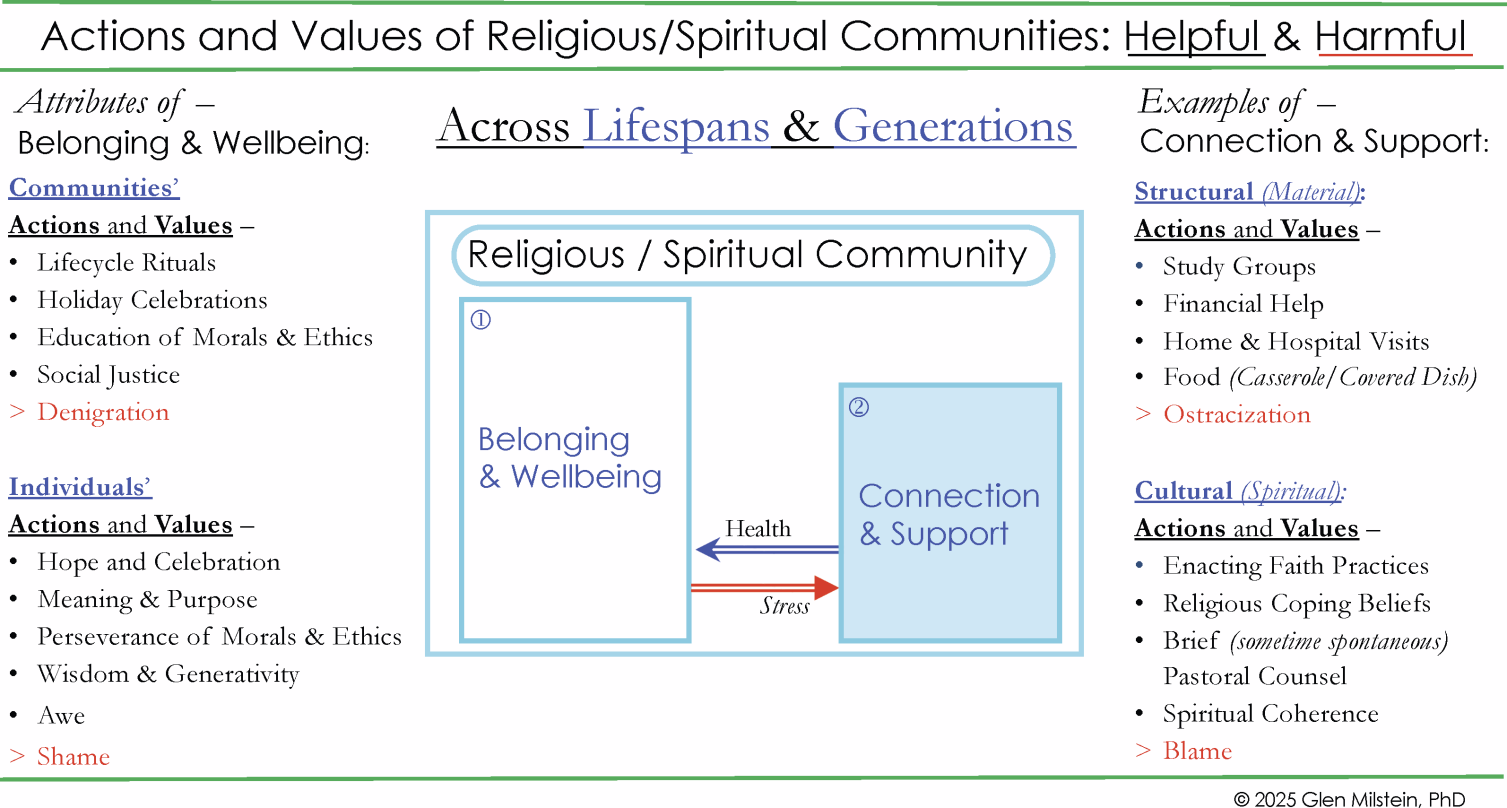
Actions and values of religious/spiritual communities: helpful & harmful.

The psychological attributes of attendance likely involve many distinct motivations and ways to experience religious services (50). Congregations invite people to become part of a community; they are spaces where congregants might enact personal and communal prayer; where there is learning and teaching. Persons celebrate ceremonial events and lifecycle rituals from birth, through school years to marriage, child-rearing and until death (23, 76–78). Since people go to services for so many reasons, attendance may be the source of widespread benefits exactly because different people benefit in different ways at different stages of life (10, 48). What they do share are the benefits – and possible harm – of attendance in a locatable physical space (**Figure 3**).

##### 2.3.1.2 SFBOs are locatable

In order for MHSOs to offer care as early and effectively as possible, to target prevention initiatives at developmentally sensitive periods, and to sustain recovery via community, collaboration will necessitate locating capable and caring partners (1). Religious congregations and other SFBOs are usually housed in physical spaces, and as noted, there are over 300,000 SFBOs’ ***doors to knock on***. Twenty-first century web-based technology makes it easy to create maps of the locations of SFBOs near to MHSOs, as well as to record the types of public health resources they provide (food banks, childcare, after-school, housing support, Alcoholic Anonymous meetings…). Details for the use of a free web-based program to map locations and resources is found in **Appendix 1**. Another structural, public mental health advantage of MHSOs outreach to SFBOs is that this work *creates **no new infrastructure***; it bridges a continuity of collaboration between extant organizations, without necessitating any new building or capital campaigns.

While congregations may facilitate positive social determinants of health, they are not clinics. Rather, clinics (and clinicians in private practice) can provide mental health care through professional assessments and treatments. In turn, religious communities can help persons sustain recovery after treatment (79). To achieve successful outreach to connect community support and clinical treatment (**Figure 1**), MHSOs also need to understand the cultural views of SFBOs’ and individuals’ R/S as they pertain to care.

#### 2.3.2 Cultural bridges: spirituality and faith

In addition to hundreds of thousands of physical locations, in the United States alone, people belong to 236 religious denominations (74). SFBOs have denominational worldviews and meaning frameworks that are shared among their community members (**Figure 3**). In turn, these denominational beliefs and values guide R/S practices and relationships among adherents, which can support development of shared purpose, character strengths, and social connections (80). We have emphasised the positive psychological potency of R/S that can be woven into people’s personal spirituality, and serve as a source of hope and strength. It must also be recognised that R/S can produce negative outcomes (**Figure 3**). In families and communities R/S can devolve into negative judgments that harm persons mental health and wellbeing (81). Research also finds that, in the aftermath of painful transitions, traumas, or morally injurious events, faith can engender strains, tensions, and conflicts, which diminish people’s wellbeing and capacity for resilience (45, 82–86). Some persons’ present-day trauma is imbedded in a developmental history of rejection or other harm from their religious upbringing (32).

At other times, it is a theological vocabulary – in place of a medical vocabulary – that frames how people describe their lived experiences with mental illness (65, 69, 87). In the words of a young man with schizophrenia:

*“The biomedical model of mental illness has contributed significantly to our understanding of major mental illness, but little to true recovery. While medications may help one’s behavior become more acceptable to society, they do nothing to put one’s shattered soul back together.”* (88, p 25).

This person describes the disruptions and pain of schizophrenia in two ways. First, he describes the disruptions his illness causes between himself and others. Second, he describes how his illness interferes with his connection to himself and to an *ineffable transcendent*: as he says, to his *soul* (**Figure 2**). 

In another example, a mother whose son has schizophrenia, was asked if she thought his illness would ever be cured, she responded,

*Si Dios hace la obra, él se va a sanar, aunque los doctores digan que él va a ser así siempre* (89). *[If God performs the deed, he will get well, even though the doctors say he will always be like this.]*

For many religious persons, the belief in the possibility of a cure is an expression of their faith in the healing power of a deity, and also their spiritual motivation to provide care (**Figure 2**).

Therefore, to treat the whole person, any intervention that seeks to restore mental health and sustain recovery must also assess and possibly integrate resources, which – for most people – will include some combinations of religion, spirituality, faith and ritual practices (**Figure 2**). It is the disregard of R/S, on the part of mental health professionals, that may interfere with persons’ paths to wellbeing. As such, clinicians need to cultivate religion and spirituality (R/S) competencies (awareness, knowledge, and skills) to assess, discuss, and address religious or spiritual concerns with their patients, and to work therapeutically in response to these positive or negative experiences of R/S across individuals’ lifespans (38, 90).

## 3 Actionable recommendations

### 3.1 COPE: C*ommunity* O*utreach* & P*rofessional* E*ngagement* – a framework to bridge public mental health services with religious organizations

We built on lessons learned from previous generations (50, 91–94) to develop a collaborative public mental health framework to offer actionable strategies for the delivery of culturally competent care, through pathways of reciprocally beneficial partnerships between mental health service organizations (MHSOs) and spiritual/faith-based organizations (SFBOs). C*ommunity* O*utreach* & P*rofessional* E*ngagement* (COPE) is the result of decades of work with religion and spirituality (R/S) across a breadth of public mental health settings (21, 23, 28, 37–43, 45, 46, 80, 89, 95–110). In collaboration with experts in public health, systems science, clinical care, and theology, as well as with chaplains, community clergy, people with lived experiences of mental disorders and their families, we developed the adaptive COPE program to bridge community care and clinical treatment (**Figure 1**). COPE first acknowledges borders between persons’ community and their clinical treatment, and then builds programs that bridge clinical services of MHSOs with local resources of SFBOs in order to: a) strengthen wellbeing through community resilience; b) enhance access and effectiveness of clinical treatment; c) generate a continuum of collaborative care across MHSOs and SFBOs to sustain recovery and renew wellbeing (21, 39, 40, 111).

### 3.2 COPE has three core principles

1. Clergy and Religious Communities: have specific expert knowledge about religion & culture.
2. Clinicians and Research Scientists: have specific expert knowledge about assessment & treatment.
3. Professional Collaboration: can reduce burdens of SFBOs and MHSOs – and help more persons – through a continuum of wellbeing, support, treatment and recovery.

### 3.3 COPE has two implementation strategies: inreach and outreach

#### 3.3.1 Inreach

Works within an MHSO to first gauge the interest of administrators, clinicians, staff, and those who receive services, to include R/S as part of care. As a next step, an internal evaluation is often needed to determine how much the clinic’s staff demonstrates basic awareness, knowledge, and skills necessary to attend to the spiritual or religious aspects of clients’ lives (i.e., spiritual and religious competencies) and to determine whether the MHSO’s prevention and treatment programs sufficiently address this cultural domain (38, 104, 112).

For example, Inreach has identified the need to train clinicians and other staff in R/S competence and to include terms in the intake questionnaire which identify clients who prefer a spiritually responsive approach to their treatment (including collaboration with clergy or other faith leaders). COPE will guide supervisors and administrators to dedicate resources to ensure that clients and community members receive spiritually competent assessment and services from the MHSO. Even persons who report that they are not religious or spiritual may have been raised in a religion that had a negative effect on their current mental health. Therefore, a lifespan assessment of R/S is necessary, as earlier religious traumas may need to be a focus of persons’ clinical treatment (23, 105, 113).

**Figure 4:**
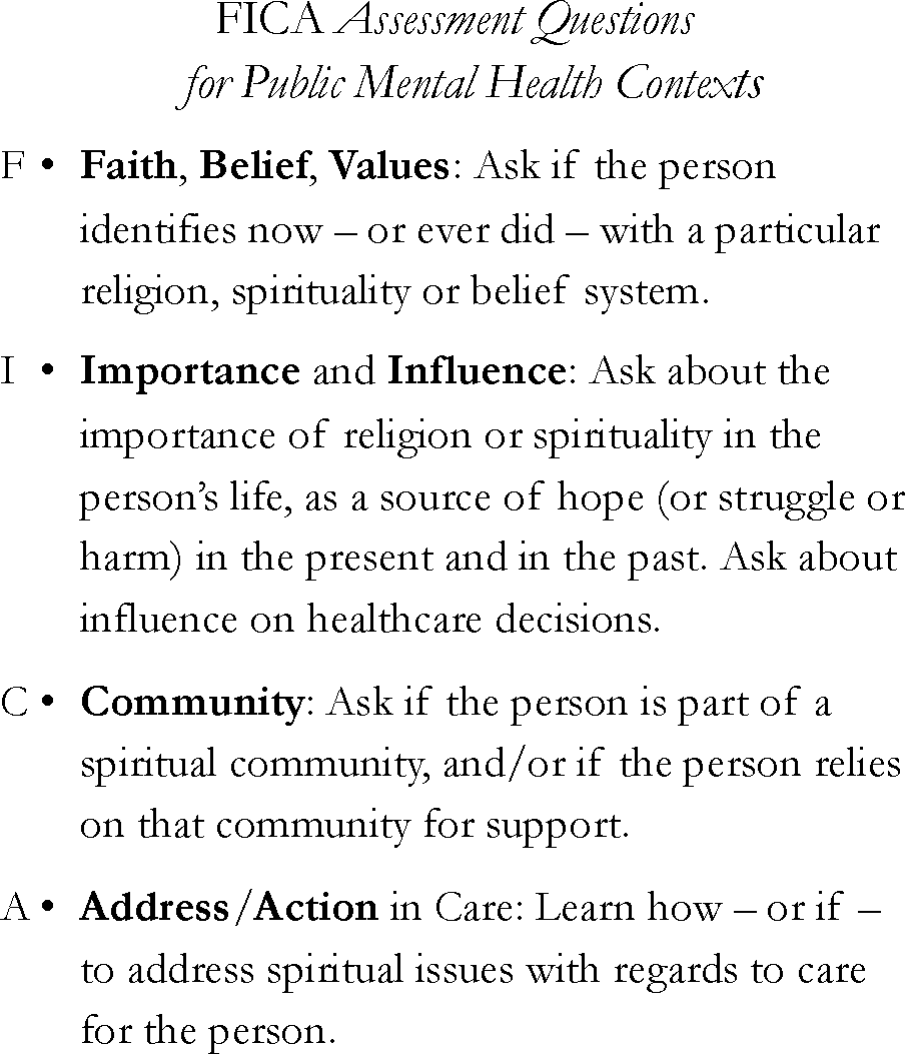
Assessment questions.

An example of a clinical assessment with demonstrated utility is the four-part FICA (Faith, Importance, Community, Action) (70, 114) (**Figure 4**). The FICA guides medical providers to facilitate conversations through open-ended questions about their patients’ R/S concerns and needs. Research has supported the acceptability, feasibility, and validity of the FICA with both patients and physicians, irrespective of the clinicians’ own R/S (115, 116). The FICA is a tool that facilitates assessment of persons’ experiences of their religious faith and spirituality – both positive and negative. It is therefore a useful guide to both individual clinical treatment, as well as to community engagement of collaborative partnerships. Other assessments are also available (28, 117–119). In **Figure 4**, we adapt the FICA assessment for public mental health contexts.

One psychotherapy training – that we have used with clinics – is the online program, *Spiritual Competency Training in Mental Health”* (SCT-MH) (120, 121). The eight-module program has demonstrated support for the effectiveness of promoting facets of foundational competence for addressing clients’ religion and spirituality (R/S) in mental health practice (112). The breadth of training modules includes instruction to, *“Distinguish between: Helpful & Harmful Spirituality”*, as well as to both *“Mobilize Spiritual Resources”* and *“Address Spiritual Problems.”* One key is to demonstrate cultural humility with persons who receive clinical care and say, “I don’t know about your faith (spirituality…). I am curious to learn from you how it helps you, or how it might cause difficulties” (122). As clergy are often concerned about how clinicians will attend to peoples’ spiritual and religious identities, this dedication to train R/S competence for clinicians is also reassuring to clergy when they consider making a referral to a MHSO. In addition, work with administrators in MHSOs also revealed the possibility to track referrals from SFBOs in the Electronic Health Record to identify possible community partners and also track the success of the COPE-based initiatives for referrals from SFBOs (28).

#### 3.3.2 Outreach

Persons from MHSOs contact SFBOs to inquire about unmet mental health needs and to initiate exploratory dialogue with MHSOs. When SFBOs have an interest, this process ideally leads to co-creating bidirectional educational presentations. Outreach and dialogues can nurture familiarity that allow for referrals. In turn, this can build trust and long-term partnerships and collaborations. We have learned that MHSOs need to designate a person to lead these outreach efforts. They are the bridge builders. Frequently they are persons with both clinical and pastoral training, and often have certification in both areas. This allows one MHSO to reach out to many SFBOs, regardless of denomination, often guided by persons who receive services. Webinars that describe these programs are found in Appendix 2. Appendix 3 provides a selection of online resources.

An example is when MHSOs teach *Mental Health First Aid* (123) courses in SFBOs, which help congregations support those members facing mental health stressors. At the same time, clergy demonstrate to clinicians the de facto care provided outside of the clinic, allowing those who return to the community from clinical treatment to have more support for sustained recovery from within the community. With this bridge of collaboration, there is burden reduced on both clinicians and clergy. There is burden reduction for clergy who know they can make a referral for a member of the congregation. There is burden reduction for clinicians when they know that there is a participant community to provide a “solid welcome” to their patient, to sustain the hard-won recovery (28, 42). To guide these initiatives and demonstrate the interdependence and opportunities for prevention, treatment and sustained recovery we developed the COPE framework.

### 3.4 COPE bridges community care with clinical treatment and maps collaborative flows

COPE: C*ommunity* O*utreach* & P*rofessional* E*ngagement* (**Figure 1**) diagrams the dynamic flows of relationships between SFBOs in community contexts and MHSOs in clinical contexts (13, 15, 124–126). **Figure 1** is a snapshot of the epidemiology of mental health in a society. The reducing sizes of the rectangles represent the smaller number of persons who pertain to the category. This is to be expected as the increased shading represents increased dysfunction caused by more debilitating forms of mental disorder and substance abuse (8, 14, 79).

The larger, Community rectangle encompasses two categories. The first category is how most people spend most of their time: (1) Here we experience Belonging – to a community, to a family, to a home (127). This generates Wellbeing (10, 117, 128–133). Next is: (2) Connection to community, which offers us hope and Support in times of stress (134). 

The smaller, Clinic rectangle, encompasses the next two, more-shaded rectangles. These show the place(s) for persons who need to receive clinical treatment(s) in response to dysfunction. For most persons, this is: (3) Outpatient Treatment. Some persons require: (4) Intensive (or Inpatient) Treatment for their severe or chronic mental illness or substance abuse. 

The greatest challenge for persons with chronic mental disorders and chronic substance abuse is to sustain recovery. As noted in rectangle (5) Community-Engaged Collaborative Care: it is when persons can avail themselves of both Community and Clinic that that they can best Sustain Recovery.

Some religions’ stigma about the veracity of mental illness makes it a challenge for people to be willing to seek clinical treatment. In turn, few clinical treatments guide clinicians on how to return people to community connections that provide support and belonging (135, 136). The COPE framework identifies the work required to create programs that will bridge persons sustained movement between community and clinic. This work requires MHSOs to support their staff who work as community outreach bridge builders as they seek partnerships with community SFBOs.

COPE maps two directions of flows. The red arrows that flow from left to right demonstrate worsening conditions that devolve into three flows of: *Stress*, *Dysfunction*, and *Severity/Chronicity*. However, most stress occurs and – ideally – is resolved in our homes and communities. We experience *Stress*, which leads us to seek Community Support. We then experience our Health Flow, with a return to Wellbeing and no need for clinical treatment.

It becomes necessary to crossover beyond our Community to receive Clinical Treatment when our difficulties flow (or may flow) to *Dysfunction* (2, 137). Preferably, we have access to care and can receive outpatient treatment to resolve our dysfunction. The *Severity/Chronicity* flow of *s*ome mental illnesses and some substance abuse may require Intensive or even Inpatient Treatment. There can be fruitful collaboration in these settings between clinicians and hospital chaplains (100, 138). The goal for COPE is to engage as many settings and stakeholders to work together as possible. The blue arrows, flowing from right to left, represent improved conditions cultivated by clinical interventions and community supports, which can evolve into flows of: Reintegration, Recovery, and Health. Recovery and wellbeing will best be sustained by community-engaged collaborative care.

### 3.5 Bereavement pathways mapped by COPE

The universal human experience of loss is frequently the cause of profound stress (139, 140). Human bereavement provides robust paradigms to elucidate the dynamic flows of the continuities of care mapped by COPE. **Figure 3** is a diagram of the first two categories of COPE: Belonging and Connection, which in turn provide Wellbeing and Support. These are found in communities, not clinics.

**Figure 3** illustrates the *normative* flows of the vast majority of persons in response to grief (139, 141). Here we see how roots in a community provide a sense of Belonging through shared actions and values across lifespans *(and generations)*, which support and affirm Wellbeing. When people lose someone close to them, many reach out to their faith leaders and communities for material, emotional, and spiritual support (142). In response to this *Stress* Flow, faith leaders and fellow congregants of SFBOs may offer the structural (*material*) Support such as, visits, financial help and even prepared meals. They may also offer the cultural *(spiritual)* Support to enact mourning rituals, which may help to re-establish connection, intelligibility, and belonging (141). For most persons these rituals are practiced from their youth to their old age and provide a spiritual coherence that can engage hope (23, 143–145). In most bereavement situations – through a period of mourning – these supports can return persons to Wellbeing without any involvement of clinicians: the Health Flow. A crucial caveat is that grieving can also be a time when the negative attributes of religion can energise judgments of *shame* and *blame* from family or community or within oneself, leading to spiritual incoherence and worsened mental health (32).

Returning to the full COPE diagram in **Figure 1**, some persons’ bereavement might instead lead to persistent distress or *Dysfunction* Flow that warrants clinical care. In those cases, family, friends, or clergy could recognize these warning signs and refer the person to a trusted clinic or provider. In turn, this Outpatient Treatment will ideally promote a Recovery Flow that returns people to Support and Wellbeing in their communities. However, some people require more assistance in their recovery journey. In particular, we have learned that those who are typically most in need of Intensive clinical care often have complex histories of trauma over the life span, possibly with addiction, and other issues. In such cases, persons’ *Severity/Chronicity* Flow may pose harm to themselves or others and lead to needed Intensive (or Inpatient) Treatment. When persons are discharged, **Figure 1** illustrates how Community Engaged Collaborative Care enacted across people receiving services as well as their clinicians, peers, families, and faith leaders can then limit social isolation to engage hope, promote moral agency, facilitate Reintegration Flow, sustain Recovery Flow and a Health Flow that returns to Wellbeing and Sustained Recovery (146).

### 3.6 COPE: case studies^1^

The COPE framework has been adopted in multiple locations, and informed work at others. Here, we share three initiatives with clinical case examples. Two programs were built based on COPE, the third built their program independently while working collaboratively with our team. In our work, we consistently see that SFBOs have frequent experiences with mental health and substance problems among their congregants, and equally evident is that MHSOs know the importance of R/S among the people they serve. What SFBOs and MHSOs need is a bridge to bring them together (**Figure 1**). Below are three examples.

#### 3.6.1 Denver, Colorado

The Director of Clinical Services at a comprehensive mental health center in Denver, CO reached out to the first author in order to best respond to interest from her staff on how to address the R/S concerns of the persons under care in their clinics. A survey of the staff and those receiving services quantified the significant interest in R/S as an aspect of care. Using an earlier version of the COPE framework (21), they worked with their new *Director of Faith and Spiritual Wellness* (DFSW) to launch the program. The DFSW was trained both as a minister and in mental health. Inreach and Outreach programming was developed. For Inreach programs, the clinic staff was guided to add R/S questions to both the intake and treatment plan forms; discussion groups were held to study the varieties of religious experiences of both the staff members, and the people receiving care at the clinic. The DFSW reached out to local congregations in the community that had demonstrated interest in how to use mental health resources in the care of persons in their communities. This bridge had immediate results: from congregations, the DFSW received requests for training on mental health issues and interventions, which led to providing regular programming in congregations on mental health first aid. The clinic also started a peer-support spirituality group. More bridges were built between the Denver clinic and the congregations: the clinic organized a half-day seminar to bring together clergy, clinicians and consumers. The heart of the program was a panel led by persons in treatment for mental illness. Each speaker was accompanied by both one’s therapist and one’s clergy. The speakers spoke about their struggles to bring these two resources of their life to become complementary. Their therapists said that since R/S inquiry was not part of their training, it had not been part of their therapy work, until these clients brought it up. Their clergy spoke of how their early views were that persons did not need mental health care, they needed to pray harder, until these parishioners confirmed a need for both R/S and clinical care to have shared presence in their lives. A review of feedback from the conference demonstrated that both clergy and clinicians in attendance viewed the COPE framework as a way to guide access to the others’ expertise; furthermore, persons of different traditions were open to receive care inclusive of religious resources. Most people felt the COPE framework delivered at the seminar not only bridged the distance between local clergy and mental health professionals, it had given all a common language with which to explore their shared interests (28).

#### 3.6.2 Mobile, Alabama

For three years we implemented COPE in the Vets Recover (VRR) clinic in Mobile, Alabama, which specializes in serving veterans, first responders and their families. VRR has Inpatient, Outpatient, Peer-Support and Community Integration services. The COPE Coordinator (CC) – working for Community Integration – was part of Inreach training of clinicians and led an extensive outreach program with presentations to churches that have included *Mental Health First Aid*, *Trauma Informed Care*, *Black Men and Mental Health* and presentations on the *Bible and Wellbeing*. The CC is both an ordained pastor and certified counsellor (and third author). The CC also worked with local ministries who serve unhoused people. One man served by the ministry worked with the CC, and the man requested a referral to the outpatient clinic. Through the therapy the person worked to stop substance use, to return to his vocation as a welder and to become a member of a local congregation. The CC reports that the way to build the necessary trust for healing with this person, was to approach the man with sensitivities steeped in ethnicity and religiosity. It was crucial to the man that his religion be respected, and as the clinic had invested in understanding R/S, the man’s care benefited from the connection between his faith, his clergy and his mental health caregiver that the CC facilitated.

#### 3.6.3 Fort Washington, Pennsylvania

Intersect is a program within Access Services, a comprehensive mental health center. They describe themselves as, “Supporting people at the intersection of faith and mental health.” Intersect has developed an independent program within Access Services. They train across the centre’s programs, do workshops at local congregations and in neighbouring cities. They have also organized a County Multi-Faith Coalition. One example of the flow of how their integrated system works is the case of a man enrolled in their psych rehab program. He took part in a spirituality group developed and led by the Director of Intersect (the fifth author), who is both an ordained pastor and social worker. Through the group the man – who had been estranged from his religion due to earlier trauma – expressed his wish to find a congregation. The Director contacted a minister he knew and the minister met with the man and the Director and then invited the man and his wife to come to services. The couple attended regularly. After some time, the man had a crisis, locked himself in the bathroom and threatened suicide. The wife called the minister. The minister called the Director who – with the minister on the line – called the mobile crisis team who were able to bring the man to treatment. Because the couple were comfortable and familiar with the minister and had never hidden the man’s mental illness, they were also comfortable returning to the social infrastructure of their recently joined church community. Sometime later the man had another crisis and, again, the wife called the minister and this time, the minister called mobile crisis directly. A bridge was built and was travelled across.

### 3.7 Future outcome research

Looking ahead, strategic and inter-disciplinary research will be needed to evaluate the feasibility, acceptability, and effectiveness of COPE as a guiding framework for mental health service organizations (MHSOs) who seek to partner with religious congregations and other spiritual/faith-based organizations (SFBOs) to engage the social infrastructures and determinants of mental health care (147, 148). Researchers might begin by considering these basic questions for varying stakeholders in their communities:

1. What are the mutual (or divergent) interests for collaboration among faith-based members and leaders, mental health care providers, people who receive care, their families, as well as civic leaders?
2. What are the community facilitators and barriers to COPE implementation?
3. What are the most effective strategies for facilitating Outreach and Inreach activities within MHSOs?
4. What are the impacts of COPE implementation on the accessibility and outcomes of clinical care?

By spearheading rigorous research on COPE to address these types of questions, MHSOs can identify resources and develop programs for training and aligning their clinical procedures to engage the role of R/S in the lives of their clientele (Inreach) along with evidence-based strategies for building partnerships with SFBOs in their communities (Outreach). Such efforts could provide the evidence base to support an increase in the number of MHSOs with a commitment to spiritually competent care and collaboration with clergy and faith leaders in ways that could strengthen community resilience, increase access to clinical care, and improve outcomes of clinical care and wellbeing in poorly resourced – as well as well-resourced – communities.

## 4 Discussion

The primary purpose of public mental health is to promote wellbeing. People are in preventable pain, which is in part due to the loss of social connections brought on by ever fewer and less vibrant social infrastructures (47, 49). WHO and SAMHSA recognize that spiritual/faith-based organizations (SFBOs) are prevalent and locatable social infrastructures that could partner productively with mental health service organizations (MHSOs) to better address mental health distress.

COPE (C*ommunity* O*utreach* & P*rofessional* E*ngagement*) is a framework that facilitates interaction between MHSOs and SFBOs, and contributes to community wellbeing through collaborative care. This collaborative bridge between MHSOs and SFBOs informs mental health professionals so that – when salient – they may create a more substantive role for religion and spirituality (R/S) in the treatment of those seeking their care. COPE also trains clinicians to assess for how religion could be a negative source of trauma for persons they serve.

COPE guides MHSOs to offer SFBOs training on public mental health responses to mental health needs and how to set up clinical support for clergy facing mental health issues in their congregations. The connection between community care and clinical treatment may benefit clients and congregants directly: SFBOs can help persons find clinical treatment, a public mental health collaboration with SFBOs for persons in recovery from inpatient treatment, may serve as the support system they need as they integrate from intensive clinical care to sustained recovery and wellbeing in community.

Every day, SFBOs can generate hope. Hope that is nurtured through belonging to community, which supports both wellbeing and sustained recovery. COPE is a framework to build bridges of hope between public mental health and religion.

## Footnotes

1 **Appendix 2** has links to three COPE webinars that cover more case examples and also more of the theory behind the COPE framework. Four of this paper’s authors teach in the webinars.

## Appendix 1 COPE: Building Bridges Map Instructions

1. Create COPE Asset Map in Google Sheets
  - Example: https://docs.google.com/spreadsheets/d/1maSfF_RT6a2LSjRk_MTwirL82CX6XIc7/edit?usp=sharing&ouid=113154468199152454193&rtpof=true&sd=true
  - Fields to include: Existing Connection, Name of Church, Religious Affiliation, Denomination, Location, Address, Active Website with Link, Contact Information, Phone Number, Email, Food Resource, Counseling Resource, Housing Resource.
2. Google search to fill in R/S Asset Map.
   > Complete R/S Asset Map using Google. Look for church websites and/or social media pages. Include the church if it has an active website and at least one email address to reach out to the institution.
3. Call to further fill in the R/S Asset Map.
   > Further fill out the R/S Asset Map by calling the phone number listed on the church website.
4. Create COPE Building Bridges Map in Google Maps.
  - Example: https://www.google.com/maps/d/u/0/edit?mid=1Cpub66kfu0OD_pcH6wBwyAKEyCJw6f4&usp=sharinghttps://www.google.com/maps/d/u/0/edit?mid=1Cpub66kfu0OD_pcH6wBwyAKEyCJw6f4&usp=sharing
  - Go to Google My Maps and select “Create a New Map.”
5. Edit map title & description.
   > Add the map title and description. Save information.
6. Add layers into Map
   > Select “Add layer” _and then “Edit layer name” _to rename it to the categories from the R/S Asset Map (Congregations, Other FBOs, Mental Health Service Organization, Healthcare Organization, Food Resource, Counseling Resource, Housing Resource).
7. Type the address in the search bar. Press enter. Click “add to map.”
8. Edit the data table affiliated with the layer.
  - Select the three vertical dots next to the name of the layer. This will open the data table. Insert and name columns as needed based on the layer.
  - Refer to the R/S Asset Map to determine what columns to add to the layer (Point of Contact, Denomination, Solid Welcome, Website).
9. Consider changing the color of each layer to better distinguish them from one another.
10. Drop pins into Map for each Church identified in the R/S Asset Map.
   > Search for Church in the Search Bar. A pin will be dropped into the location of the Church on the Map. Click the + button to add the Church into your Map. Drag the Church under the appropriate layer. Note that some Churches may be found under multiple layers, depending on the services provided.
11. Open each Church to add content to the data table.
   > Select the pencil icon. Input the content into the data table from the R/S Asset Map. Once all content is added to the Map, you can turn on and off the layers by selecting the check box to the left of the name of the layer.
12. Set the privacy features and sharing capabilities of the Map.
   > Select “Share” and edit how to share it based on your needs.

## Appendix 2 – Online COPE Webinars:

U.S. Health & Human Services Center for Faith & Opportunity Initiatives

Milstein, G., Steinberg, T., Eckert, D. & Moister, D. (2020, August). *Meaning & Meaningfulness: Building Bridges across Borders of Religion & Mental Health Care.* Webinar for the series, Treating The Whole Person: Faith and Mental Health Professionals Partner to Address Mental Illness, sponsored by the U.S. Health & Human Services Center for Faith & Opportunity Initiatives, (*https://www.youtube.com/watch?v=ceBj7v48Tso*)

Cambridge University, Faculty of Divinity

Milstein, G. & Davison, A. (2021, October 26). *The utility of the ineffable: Lifespan development and public mental health applications of religion.* A Paper Presented to the Interreligious Relations Seminar, Faculty of Divinity, University of Cambridge, UK (*https://www.youtube.com/watch?v=2ei_IukT5S4*)

SAMHSA – Southeast Mental Health Technology Transfer Centers (MHTTC)

Milstein, G., Currier, J. & McKnght, M. (2024, February). *Addressing Spirituality in the Clinic & Community.* Webinar for the series, Treating The Whole Person: Faith and Mental Health Professionals Partner to Address Mental Illness, sponsored by the U.S. SAMHSA – Southeast Mental Health Technology Transfer Centers (MHTTC)

*https://www.youtube.com/watch?v=toScM79x0Ns*

*https://mhttcnetwork.org/products_and_resources/on-demand-addressing-spirituality-in-the-clinic-community-strategies-for-expanding-mental-health-care-sustained-recovery/*

*https://mhttcnetwork.org/event/addressing-spirituality-in-the-clinic-community-strategies-for-expanding-a-continuum-of-mental-health-care-and-sustained-recovery/*

*https://www.samhsa.gov/faith-based-community-engagement*

## Appendix 3 – A Selection of Online Resources in the United States:

• Mental Health Service Organizations (MHSOs) –

 > American Psychiatric Association –     http://www.psychiatry.org/faith

  + Mental Health: A Guide for Faith Leaders – https://www.psychiatry.org/File%20Library/Psychiatrists/Cultural-Competency/faith-mentalhealth-guide.pdf

  + Mental Health: A Guide for Faith Leaders, Quick Reference – https://www.psychiatry.org/getmedia/65c13884-1ff9-45ae-b1b6-ac18fefbf82d/Mental-Health-A-Guide-for-Faith-Leaders-Quick-Reference.pdf

 > U.S. Dept. of Veterans Affairs Mental Health and Chaplaincy Learning Collaborative: https://www.mirecc.va.gov/IMH/CollaborativeCare.asp

 > Nathan Kline Institute for Psychiatric Research

  + A Pastoral Education Guide: Responding to the Mental Health Needs of Multicultural Faith Communitieshttp://nkiccase.wpengine.com/wp-content/uploads/2016/01/mentalhealthclergyguide101711A.pdf .

  + A Pastoral Education Workbook: Responding to the Mental Health Needs of Multicultural Faith Communitieshttp://nkiccase.wpengine.com/wp-content/uploads/2016/01/mentalhealthworkbook111511.pdf

 > National Alliance on Mental Illness Faith Net www.nami.org/namifaithnet

  + NAMI_2024_FaithNet Awareness Bulletin https://www.nami.org/wp-content/uploads/2023/10/AwarenessBulletin.pdf

 > Humanitarian Disaster Institute: https://www.wheaton.edu/academics/academic-centers/humanitarian-disaster-institute/

  + Spiritual First Aid, Community: https://www.spiritualfirstaid.community/

  + Spiritual First Aid: https://www.spiritualfirstaid.org/

 > Intersect of Access Services: https://www.accessservices.org/intersect/

  + List of programs: https://www.accessservices.org/wp-content/uploads/2024/08/intersect_booklet_faithcommunities_aug142024.pdf

• Spiritual/Faith-Based Organizations (SFBOs) –)

 > Pathways to Promise Interfaith Ministries: www.pathways2promise.org

 > Mission Peak Unitarian Universalist Congregation

  + Mental Health Ministry https://mpuuc.org/mhm/

  + Caring Congregation Program https://mpuuc.org/mhm/mental-health-programs/caring-congregation-program/

 > First Corinthian Baptist Church:

  + HOPE (Healing On Purpose and Evolving) Center https://hopecenterharlem.org/

 > The Potter’s House

  + The Center for Counseling and Behavioral Health https://www.thepottershouse.org/ministries/counseling-ministry/

• Denominational Resources:

 >  Christianity:

  + United Church of Christ Mental Health Network: https://www.mhn-ucc.org/

    — WISE (Welcoming, Inclusive, Supportive, and Engaged) Congregations Program: https://www.mhn-ucc.org/becoming-wise/

  + National Catholic Partnership on Disability: https://www.ncpd.org/disability-ministry/mental-illness

  + Presbyterian Church U.S.A – https://pcusa.org/about-pcusa/agencies-entities/interim-unified-agency/ministry-areas/mental-health-ministry

  + United Methodist Ministries in Mental Illness: http://www.umc.org/what-we-believe/ministries-in-mental-illness

  + The Church of Jesus Christ of Latter-day Saints: https://www.churchofjesuschrist.org/get-help/mental-health?lang=eng

 > Islam https://muslimmentalhealth.com

 > Judaism: https://prizmah.org/knowledge/resource/jewish-mental-health-organizations

  + Interdenominational – Blue Dove: https://thebluedovefoundation.org/

  + Orthodox – https://neshamos.org/
